# Supplementary material for: Comparative analysis of pattern-triggered and effector-triggered immunity gene expression in susceptible and tolerant cassava genotypes following begomovirus infection
Source: PLoS One. 2025 Jun 4;20(6):e0318442. doi: 10.1371/journal.pone.0318442 (PMC12136462; doi:10.1371/journal.pone.0318442)
Supplement: S1 Table — SI refers to SACMV symptom index. (DOCX) [file pone.0318442.s001.docx]

**S1 Table.** CMD-type resistance and cassava genotypes symptomatic scoring determined using a scale of 1–5. SI refers to SACMV symptom index.

| **Genotype** | **CMD-type** | **Response (SACMV)** | **SI 32 dpi** | **SI 67 dpi** |
| --- | --- | --- | --- | --- |
| TMS98/0505 | CMD3 | Resistant | 1 | 1 |
| TME3 | CMD2 | Tolerant, recovery | 3 | 1 |
| T200 | CMD2 | Susceptible | 3 | 3 |
| Ukulinga 9 | CMD2 | Susceptible, delayed | 1 | 3 |
